# Substrate-bound outward-open structure of a Na^+^-coupled sialic acid symporter reveals a new Na^+^ site

**DOI:** 10.1038/s41467-018-04045-7

**Published:** 2018-05-01

**Authors:** Weixiao Y. Wahlgren, Elin Dunevall, Rachel A. North, Aviv Paz, Mariafrancesca Scalise, Paola Bisignano, Johan Bengtsson-Palme, Parveen Goyal, Elin Claesson, Rhawnie Caing-Carlsson, Rebecka Andersson, Konstantinos Beis, Ulf J. Nilsson, Anne Farewell, Lorena Pochini, Cesare Indiveri, Michael Grabe, Renwick C. J. Dobson, Jeff Abramson, S. Ramaswamy, Rosmarie Friemann

**Affiliations:** 1 Department of Chemistry and Molecular Biology, University of Gothenburg, Box 462, S-40530 Gothenburg, Sweden; 2 Centre for Antibiotic Resistance Research (CARe) at University of Gothenburg, Box 440, S-40530 Gothenburg, Sweden; 3 Department of Life Sciences, Imperial College London, Exhibition Road, London, South Kensington SW7 2AZ UK; 4 Membrane Protein Lab, Diamond Light Source, Harwell Science and Innovation Campus, Chilton, Oxfordshire OX11 0DE UK; 5 Rutherford Appleton Laboratory, Research Complex at Harwell, Didcot, Oxfordshire OX11 0FA UK; 6 Biomolecular Interaction Centre and School of Biological Sciences, University of Canterbury, Private Bag 4800, Christchurch, 8041 New Zealand; 7 Department of Physiology, David Geffen School of Medicine, University of California, Los Angeles, CA 90095-1751 USA; 8 Department DiBEST (Biologia, Ecologia, Scienze della Terra) Unit of Biochemistry and Molecular Biotechnology, University of Calabria, Via P. Bucci 4C, 87036 Arcavacata di Rende, Italy; 9 Cardiovascular Research Institute, Department of Pharmaceutical Chemistry, University of California, San Francisco, CA 94158 USA; 10 Department of Infectious Diseases, Institute for Biomedicine, Sahlgrenska Academy, University of Gothenburg, Box 440, S-40530 Gothenburg, Sweden; 11 Centre for Analysis and Synthesis, Department of Chemistry, Lund University, POB 124, S-22100 Lund, Sweden; 12 Department of Biochemistry and Molecular Biology, Bio21 Molecular Science and Biotechnology Institute, University of Melbourne, 30 Flemington Road, Parkville, VIC 3010 Australia; 13 The Institute for Stem Cell Biology and Regenerative Medicine (InStem), GKVK Post, Bangalore, 560065 Karnataka India; 14 Department of Structural Biology, School of Medicine Stanford University, 299 Campus Drive West Stanford, Stanford, CA 94305-5126 USA

## Abstract

Many pathogenic bacteria utilise sialic acids as an energy source or use them as an external coating to evade immune detection. As such, bacteria that colonise sialylated environments deploy specific transporters to mediate import of scavenged sialic acids. Here, we report a substrate-bound 1.95 Å resolution structure and subsequent characterisation of SiaT, a sialic acid transporter from *Proteus mirabilis*. SiaT is a secondary active transporter of the sodium solute symporter (SSS) family, which use Na^+^ gradients to drive the uptake of extracellular substrates. SiaT adopts the LeuT-fold and is in an outward-open conformation in complex with the sialic acid *N*-acetylneuraminic acid and two Na^+^ ions. One Na^+^ binds to the conserved Na2 site, while the second Na^+^ binds to a new position, termed Na3, which is conserved in many SSS family members. Functional and molecular dynamics studies validate the substrate-binding site and demonstrate that both Na^+^ sites regulate *N*-acetylneuraminic acid transport.

## Abstract (web summary)

Sialic acid transporters (SiaT) are required for sialic acid uptake in a number of human pathogens and are of interest as targets for antimicrobial drug development. Here the authors present the substrate bound SiaT structure from the uropathogen *Proteus mirabilis* and provide insights into the mechanism of sialic acid transport.

## Introduction

Many pathogenic and opportunistic bacteria have evolved the ability to scavenge and metabolise sialic acids^1,2^—a large family of nine-carbon acidic monosaccharides prevalent in mucus rich environments^3^. In mammals, sialic acids are primarily found at the terminal end of cell surface glycoconjugates, where they mediate a diverse array of biological functions^1,2,4,5^. To facilitate the import of scavenged sialic acids, bacteria that colonise sialylated environments deploy specific transporters, including those from the ATP-binding cassette (ABC)^6^, tripartite ATP-independent periplasmic (TRAP)^7,8^, major facilitator superfamily (MFS)^9^ and sodium solute symporter (SSS)^10^ transporter families (reviewed by North et al.^11^). Once imported into the cytoplasm, bacteria utilise host-derived sialic acids either for molecular mimicry, where sialic acid is incorporated into their surface glycoconjugates, or use sialic acids as sources of carbon, nitrogen and energy^1,12,13^. Despite a growing understanding of the catalytic steps involved in the cleavage of sialic acids from the host cell surface and subsequent cytoplasmic processing^2,3^, little is known about the molecular determinants of import. Disruption of the genes encoding sialic acid transporters impairs outgrowth of *Salmonella enterica* serovar Typhimurium and *Clostridium difficile* during post-antibiotic expansion^14^ and of *Escherichia coli* during intestinal inflammation^15^.

The uropathogen, *Proteus mirabilis*, catabolises host-derived sialic acids as a source of energy; the genes required for the transport and degradation of sialic acids are encoded within the *nan* operon^10^. The sialic acid transporter of *P. mirabilis* (SiaT) is a secondary active transporter of the SSS family^10^, which use the Na^+^ electrochemical gradients as the driving force for the uptake of extracellular substrates.

The first structural representative of the SSS family is the sodium galactose transporter from *Vibrio parahaemolyticus* (vSGLT)^16^. The vSGLT structure consists of 14 transmembrane (Tm) helices, where the substrate and Na^+^ binding sites are located centrally within two five-helix inverted repeats, known as the LeuT-like fold. This fold is shared among many sodium-dependent symporters, which operate through the alternating access mechanism^16–19^.

The number of sodium binding sites for transporters that adopt the LeuT fold varies. LeuT^20^ and the *Drosophila melanogaster* dopamine transporter (dDAT)^21^ possess two sodium binding sites (Na1 and Na2), where the Na^+^ of Na1 directly coordinates the substrate in LeuT. The betaine symporter (BetP)^22,23^ has the conserved Na2 binding site and a putative Na1´ binding site, which is distinct from the Na1 site in LeuT and dDAT. The structures of vSGLT^16^ and the benzyl-hydantoin transporter (Mhp1)^24^ identified a single conserved binding site (Na2). The Na^+^/substrate stoichiometry differs within SSS family members. For vSGLT^16,25^, human sodium glucose transporter 2 (hSGLT2)^26^ and Na^+^/proline transporter (PutP)^27^, the stoichiometry is 1:1, while for hSGLT1^26^ and Na^+^/I^−^ transporter (NIS)^28^, it is 2:1.

Here we report the high-resolution (1.95 Å) substrate-bound outward-open structure and a functional and biophysical characterisation of SiaT. The structure is in complex with the sialic acid *N*-acetylneuraminic acid (Neu5Ac) and two Na^+^ ions. One Na^+^ binds to the conserved Na2 site, whereas the second Na^+^ binds a new position that we term Na3. Our results inform how secondary active transporters harness additional energy from ion gradients by changing their stoichiometry, thus it might be possible to pharmacologically exploit differences in this mechanism between SSS family members and other transporters with the LeuT fold.

## Results

### Overall structure and the sialic acid binding site

**Fig. 1:**
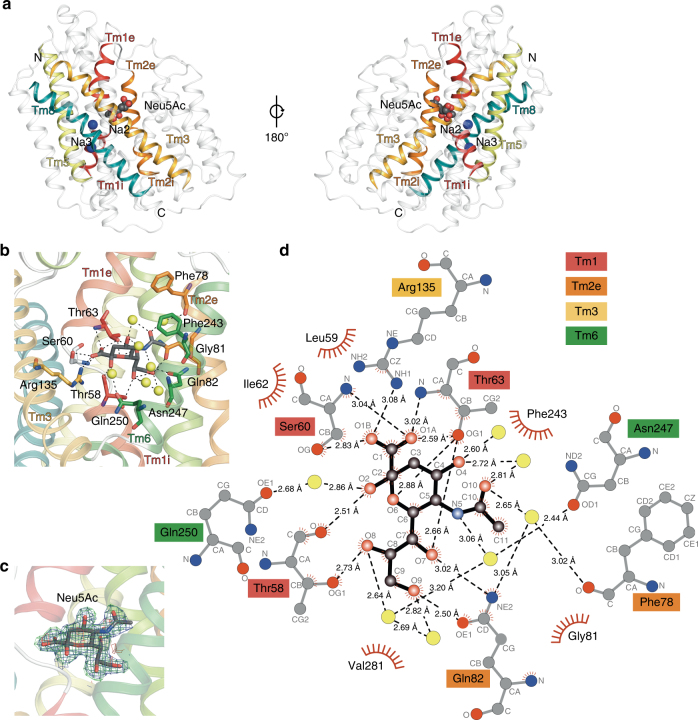
Overall architecture and the sialic acid binding site of SiaT. **a** Side-view of SiaT in the membrane plane. Transmembrane helices that coordinate with Neu5Ac and Na^+^ ions are depicted in colour, while the remaining helices are coloured in white. Neu5Ac is shown as grey spheres coloured by atom type and Na^+^ ions are shown as blue spheres. **b** Neu5Ac forms hydrogen bonds with Thr58 (Tm1), Thr63 (Tm1), Ser60 (Tm1) and Gln82 (Tm2) and a salt bridge with Arg135 (Tm3). Neu5Ac also forms water-mediated hydrogen bonds with Gln82 (Tm2), Asn247 (Tm6), Gln250 (Tm6) and Phe78 (Tm2). **c** Omit maps for Neu5Ac generated by removing respective ligands from the X-ray structure followed by refinement. The 2*F*_o_ − *F*_c_ electron density map is contoured at *lσ* (blue), the *F*_o_ − *F*_c_ map is contoured at 3*σ* (green) and −3*σ* (red). **d** The SiaT–Neu5Ac interaction network represented as a Ligplot^+^ diagram. Hydrogen bonds (dashed lines), hydrophobic contacts (arcs with spokes) and interacting water molecules (yellow) are shown

To gain insight into sialic acid uptake, we determined the structure of SiaT from *P. mirabilis* (Fig. 1a, Table 1, Supplementary Fig. 1 and Supplementary Movie 1). Homologues of SiaT are found in a wide range of pathogenic bacteria including *Streptococcus pneumoniae, S. enterica, Staphylococcus aureus* and *C. difficile* (Supplementary Table 1). SiaT comprises 13 transmembrane helices (Tm0 and Tm1-Tm12) with the N- and C-termini facing the periplasmic and cytoplasmic spaces, respectively. The core structural fold is formed by two inverted repeats of five transmembrane helices (Tm1-Tm5 and Tm6-Tm10), consistent with the LeuT-fold^17^ (Supplementary Fig. 1d).

**Table 1:** Data collection and refinement statistics

	SeMet-SAD_13merged_^a^	SeMet (5NV9)^b^	Native (5NVA)^c^
Data collection			
Space group	*C*2	*C*2	*P*22_1_2_1_
Cell dimensions			
*a*, *b*, *c* (Å)	130.24, 97.99, 54.74	130.59, 98.07, 54.78	48.78, 97.76, 151.69
*α*, *β*, *γ* (°)	90, 92.16, 90	90, 92.15, 90	90, 90, 90
Resolution (Å)^d^	19.93–3.87 (4.32–3.87)	78.40–1.95 (2.00–1.95)	82.18–2.26 (2.34–2.26)
*R*_sym_ (%)^d^	25.9 (31.7)	17.4 (134.1)	14.8 (139.6)
*I*/*σI*^d^	29.1 (28.6)	6.43 (1.22)	8.39 (1.39)
CC 1/2^d^	0.998 (0.997)	0.991 (0.452)	0.99 (0.388)
Completeness (%)^d^	99.0 (99.1)	99.2 (99.6)	96.64 (95.47)
Redundancy^d^	63.8 (64.4)	3.24 (3.28)	4.9 (4.9)
Refinement			
Resolution (Å)		78.40–1.95	82.18–2.26
No. of reflections		47,316	61,303
*R*_work_/*R*_free_		19.88/24.35	22.43/26.08
No. of atoms		3998	3834
Protein		3715	3647
Neu5Ac		21	21
Sodium		2	2
DDM		35	
Phosphate		5	
Water		220	164
*B*-factors		29.9	33.9
Protein		28.5	33.6
Neu5Ac		23.9	29.9
Sodium		27.6	32.1
DDM		47.9	
Phosphate		54.9	
Water		42.7	42.0
R.m.s. deviations			
Bond lengths (Å)		0.017	0.002
Bond angles (°)		1.85	0.51

^a^SeMet-SAD dataset was collected from 13 crystals

^b^SeMet dataset was collected from one crystal

^c^Native dataset was collected from one crystal

^d^Values in parentheses are for highest-resolution shell

The sialic acid binding site is near the centre of the protein, lined by residues from four helices (Tm1-Tm3 and Tm6) (Fig. 1b). The electron density in this site corresponds to Neu5Ac in its β-anomeric form (Fig. 1c) as seen in the *Haemophilus influenzae* periplasmic binding protein (SiaP) of the sialic acid TRAP system^29^. This is consistent with the discovery that bacteria that scavenge host-derived α-sialic acids from sialoconjugates, possess a mutarotase that catalyse the conversion to the more thermodynamically stable β-sialic acid anomer^30^. Tm1 and Tm6 adopt a distorted helical structure within the membrane bilayer at the point of contact with the substrate, which has implications for how binding drives the alternating-access mechanism^16–19^.

Eight residues and seven water molecules coordinate Neu5Ac (Fig. 1b, d). Thr58, Ser60 and Thr63 (Tm1) are involved in both side and main chain hydrogen bonding to Neu5Ac. The negatively charged carboxylate group of Neu5Ac forms hydrogen bonds to the hydroxyl and amine groups of Ser60 and Thr63 and a salt bridge with the guanidinium of Arg135 (Tm3). The presence of a basic residue in the sugar-binding pocket has been observed previously and is a common feature of sugar-binding proteins^16^. A conserved arginine in the sialic acid binding site of SiaP is essential for high affinity substrate recognition by the sialic acid TRAP transporter^31^. In sialidases and siglecs, arginine residues often interact with the carboxylate group of sialic acids^32,33^. The hydroxyl groups of the glycerol tail form hydrogen bonds with the side chain residues of Gln82 (Tm2) and Thr58 (Tm1). The acetyl amino moiety of the Neu5Ac methyl group is positioned in a region with a neutral electrostatic surface created by residues Phe78 (Tm2), Gly81 (Tm2) and Phe243 (Tm6). This is a common feature observed among interactions between viruses and sialic acid coated glycan molecules^34,35^, as well as in otherwise polar active sites of proteins and enzymes that use Neu5Ac as a substrate^29,36^. A hydration layer lies between Neu5Ac and Tm5-Tm6 with several hydrogen bonds to water molecules or water-mediated interactions with the side chain residues of Gln82 (Tm2), Asn247 (Tm6), Gln250 (Tm6) and the main chain of Phe78 (Tm2).

**Fig. 2:**
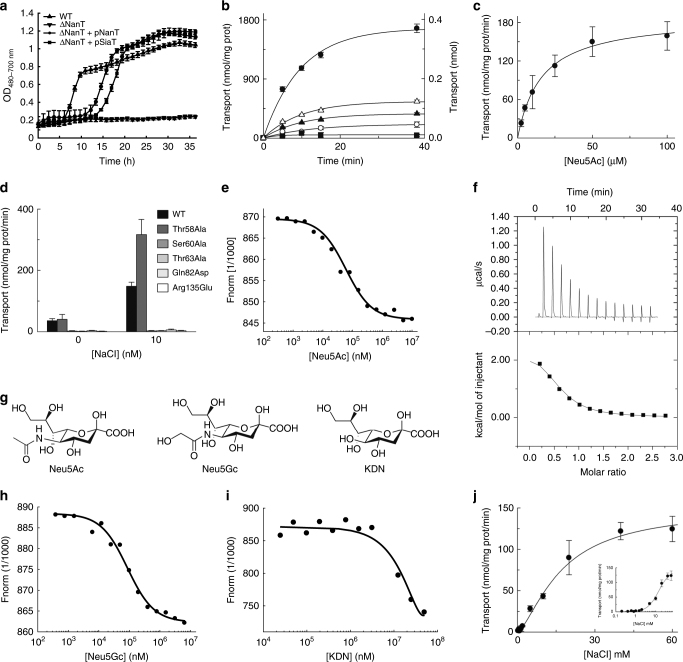
Characterisation of SiaT. **a** SiaT is able to rescue growth of *E. coli ∆*NanT on Neu5Ac as the sole carbon source. The growth lag observed for ΔNanT+pNanT and ΔNanT+pSiaT is due to IPTG induction of the T5 promoter on pNanT and pSiaT. Growth curves represent the mean of six experiments ± SEM. **b** Time course of Neu5Ac uptake into proteoliposomes reconstituted with SiaT. In (black circle, black square, white triangle, black triangle), valinomycin was added to facilitate K^+^ movement prior to transport. In (white circle), ethanol was added instead of valinomycin as a control. In (white circle, black square, black circle), 10 mM NaCl was added together with [^3^H]-Neu5Ac; in (white triangle) 10 mM KCl was used in place of NaCl; in (black triangle) no salts were used in the transport assay. In (black square), transport was measured in empty liposomes. On the left *Y*-axis, specific transport activity is reported; on the right *Y*-axis transport in empty liposomes. is reported. Uptake data were fitted in a first-order rate equation for time course plots. **c** The transport of [^3^H]-Neu5Ac in the presence of 10 mM NaCl was measured in proteoliposomes reconstituted with SiaT, with an imposed K^+^ diffusion membrane potential. Data were plotted using the Michaelis–Menten equation. **d** The kinetics of Neu5Ac transport by SiaT sialic acid binding site variants. The transport of [^3^H]-Neu5Ac with or without NaCl was measured in proteoliposomes reconstituted with wild type and mutated variants, with an imposed K^+^ diffusion membrane potential. All proteoliposome measurements (**b**–**d**) are presented as means ± SD from five independent experiments. **e** MST binding assay of Neu5Ac binding to SiaT. **f** Representative isothermal titration calorimetry raw data (top) and binding isotherm (bottom) of Neu5Ac binding with SiaT. **g** Chemical structures of *N*-acetylneuraminic acid (Neu5Ac), *N*-glycolylneuraminic acid (Neu5Gc) and ketodeoxynononic acid (KDN). **h**, **i** MST binding assay of Neu5Gc (**h**) and KDN (**i**) binding to SiaT. **j** Determination of the SiaT Na^+^ Hill coefficient. Data were plotted using the Hill equation. The inset represents the same data plotted using a log-scale for the *X*-axis to increase the resolution of low concentration data points. MST (**e**, **h**, **i**) and ITC (**f**) experiments represent the mean of three independent experiments ± SEM; for each, data from one representative experiment is shown

**Fig. 3:**
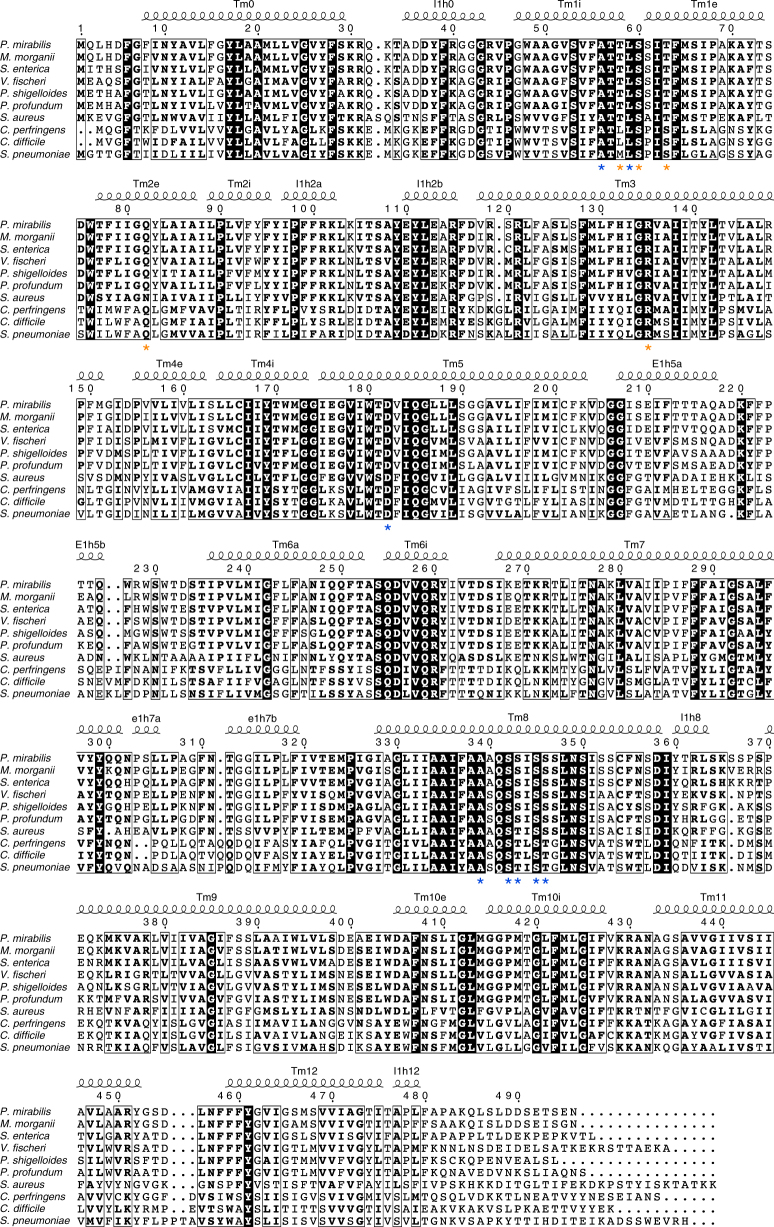
Amino-acid sequence alignment and secondary structure of *P. mirabilis* SiaT with SiaT transporters from eight additional species of bacteria. SiaT transporters from *Morganella morganii*, *S. enterica*, *Vibrio fischeri*, *Plesiomonas shigelloides*, *Photobacterium profundum*, *S. aureus*, *C. perfringens*, *Clostridium difficile* and *S. pneumoniae* are aligned. Residues are numbered according to *P. mirabilis* SiaT, and the corresponding secondary structure of this transporter is shown above the alignment, with α-helices depicted as coils. Residues highlighted with black boxes are conserved, residues implicated in sialic acid binding are highlighted below with an orange asterisk and residues involved in sodium-binding are highlighted below with a blue asterisk

To demonstrate sialic acid transport by SiaT, we first showed that SiaT rescues growth on Neu5Ac of an *E. coli* strain that lacks the endogenous NanT sialic acid transporter (Δ*nanT*) (Fig. 2a). Next, we reconstituted SiaT into proteoliposomes and measured time- and concentration-dependent uptake of [^3^H]Neu5Ac (Fig. 2b, c). This resulted in a maximal transport activity of 1800 nmol/mg protein (0.4 nmol), a *K*_M_^Neu5Ac^ of 16 ± 4 µM and a *V*_max_ of 187 ± 30 nmol/mg protein/min. In the absence of external Na^+^, the rate was reduced by 78%, similar to the value recorded with external K^+^. Five mutant transporters (Thr58Ala, Ser60Ala, Thr63Ala, Gln82Asp and Arg135Glu) were designed to disrupt substrate binding and all except Thr58Ala abolish transport (Fig. 2d), confirming their role in Neu5Ac binding. Thr58Ala exhibited twice the uptake rate of wild-type protein, and since Thr58 binds the anomeric hydroxyl of Neu5Ac, it may be involved in anomeric specificity. Thr58 is the least conserved substrate-binding residue (Fig. 3).

We confirmed Neu5Ac binding to SiaT using microscale thermophoresis (MST) (*K*_d_^Neu5Ac^ = 58 ± 1 µM) (Fig. 2e) and isothermal titration calorimetry (ITC) (*K*_d_^Neu5Ac^ = 50 ± 4 µM) (Fig. 2f).

That the *K*_d_^Neu5Ac^ is larger than the *K*_M_^Neu5Ac^ may be due to the presence of *n*-dodecyl-β-D-maltoside (DDM) detergent during the MST and ITC experiments^37^.

SiaT also binds *N*-glycolylneuraminic acid (Neu5Gc) and ketodeoxynonulosonic acid (KDN) (Fig. 2g) as determined by MST. The *K*_d_^Neu5Gc^ is 85 ± 2 µM (Fig. 2h), which is comparable to Neu5Ac binding, while KDN binding was significantly weaker (*K*_d_ > 10 mM) (Fig. 2i), demonstrating that SiaT binds different sialic acid substrates. Interestingly, SiaT has the highest affinity for Neu5Ac and Neu5Gc, which commonly occupy the terminal non-reducing position of mammalian cell surface glycoconjugates^38^.

### The sodium binding sites

**Fig. 4:**
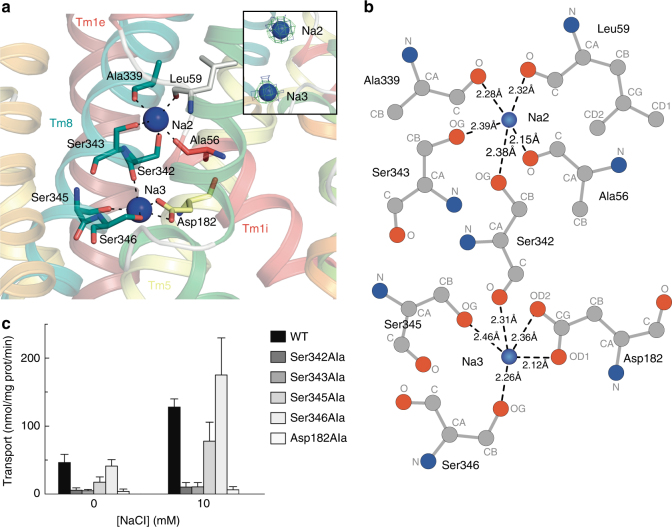
The sodium-binding sites. **a** Amino-acid residues (sticks coloured by atom type) coordinating the two Na^+^ ions (blue spheres). Inset depicts the omit map for the Na^+^ ions generated by removing respective ligands from the X-ray structure followed by refinement. The 2*F*_o_ − *F*_c_ electron density map is contoured at *lσ* (blue), the *F*_o_ − *F*_c_ map is contoured at 3*σ* (green). **b** Ligplot^+^ analysis of the SiaT and Na^+^ ion interactions. Na^+^ ion coordination is indicated by dashed lines between the atoms involved. **c** The kinetics of Neu5Ac transport by SiaT sodium-binding site variants. The transport of [^3^H]-Neu5Ac with or without NaCl was measured in proteoliposomes reconstituted with wild-type SiaT and mutated variants (Ser342Ala, Ser343Ala, Ser345Ala, Ser346Ala and Asp182Ala) with an imposed K^+^ diffusion membrane potential. Ser342Ala and Ser343Ala correspond to the Na2 site, whereas Ser345Ala, Ser346Ala and Asp182Ala correspond to the Na3 site. All proteoliposome measurements are presented as means ± SD from five independent experiments

**Fig. 5:**
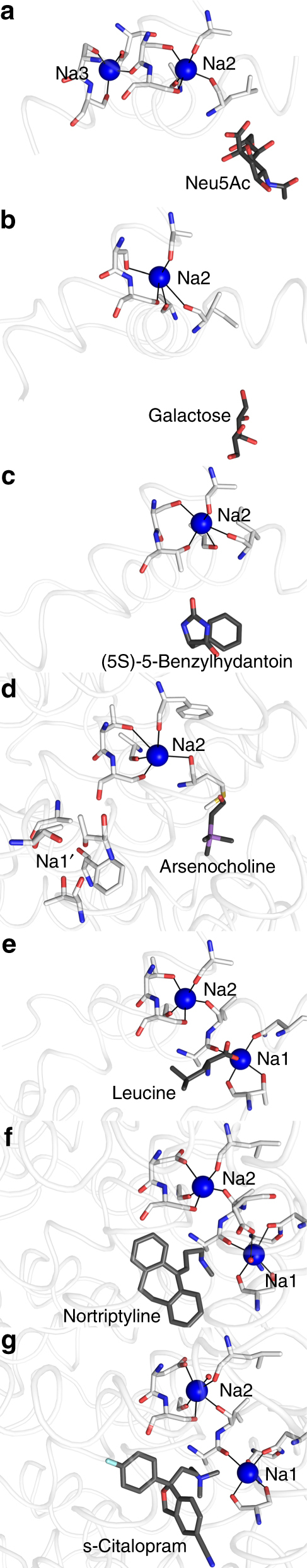
Sodium and substrate binding sites of Na^+^ transporters that adopt the LeuT fold. **a** SiaT (outward-open, pdbid: 5NV9), **b** vSGLT (inward-open, pdbid: 3DH4), **c** Mhp1 (outward-occluded, pdbid: 4D1B), **d** BetP (Asp153Gly outward-open, pdbid: 4LLH), **e** LeuT (outward-occluded, pdbid: 2A65), **f** dDAT (N-terminally truncated, EL2 deleted, Val74Aala, Val275Ala, Val311Ala, Leu415Ala, Gly538Leu, pdbid: 4M48) and **f** SERT (outward-open, Tyr110Ala, Ile291Ala, Thr439Ser, Cys554Ala, Cys580Ala, Cys622Ala, pdbid: 5I71). Substrates and amino-acid residues coordinating the Na^+^ ions are represented in sticks with carbon atoms in grey or white, respectively. Residues surrounding the putative Na1´ in BetP are shown in sticks (**d**). Oxygen atoms are red and nitrogen atoms are blue. The Na^+^ ions are represented as blue spheres

We modelled two sodium ions into the SiaT structure (Fig. 4a). One ion occupies the conserved Na2 site, which is located between Tm1 and Tm8 at a prominent kink in Tm1^19^. The second Na^+^ in SiaT occupies a unique position, which we term Na3. It is close to Na2 but is not in contact with the transported substrate nor close to either the Na1 or Na1´ sites (Fig. 5).

The Na2-binding site is ~7 Å from the substrate-binding site at the intersection between Tm1 and Tm8. The Na^+^ is coordinated by the carbonyl oxygen atoms of Ala56 and Leu59 (unwound segment of Tm1), the hydroxyl groups of Ser342 and Ser343 (Tm8), and the main-chain carbonyl oxygen of Ala339 (Tm8) (Fig. 4a, b). We demonstrated the importance of Na2 by mutating Ser342 or Ser343 to Ala, whereby both mutants lost transport activity (Fig. 4c). Na3 is 6.5 Å from Na2, towards the cytoplasm, and ~14 Å from the substrate binding site. At this position, the Na^+^ is coordinated by the main-chain carbonyl group of Ser342 (Tm8), the hydroxyl groups of Ser345 and Ser346 (Tm8), and by the carboxyl group of Asp182 (Tm5) (Fig. 4a, b). To explore the functional importance of Na3, we separately mutated Ser345, Ser346 and Asp182 to Ala. Asp182Ala abolishes Neu5Ac uptake, while Ser345Ala showed reduced uptake and Ser346Ala had slightly increased uptake (Fig. 4c). While all mutations made at the Na2 site abolish transport, the Na3 site is more nuanced, suggesting that it plays a modulatory role in the transport process. This is not surprising since many LeuT family members have only a single Na2 site, suggesting that transport is still possible if Na2 is not disturbed. Interestingly, all substrate uptake assays failed to show transport unless K^+^ was added in the presence of the passive carrier valinomycin to act as a counter ion. When gradients were imposed to create an inside negative membrane potential, transport activity was significantly stimulated, strongly suggesting that the transport cycle is electrogenic (Fig. 2b and Methods). A 1-to-1 Na^+^-substrate stoichiometry provides a neutral symport cycle, since Neu5Ac is negatively charged, but stoichiometries of 2-to-1 or higher are electrogenic supporting our claim that two Na^+^ are translocated during each cycle. In addition, sodium transport is cooperative with a Hill coefficient of 1.5 ± 0.1 (Fig. 2j), whereas Neu5Ac is not (Fig. 2c). This is consistent with the hSGLT1 Hill coefficient of 1.5 ± 0.1, which has a well-established 2-to-1 stoichiometry^39^. The sodium Hill coefficients for the Na3 site Ser mutants was determined to 1.4 ± 0.2 (S345A) and 1.2 ± 0.1 (S346A) demonstrating cooperativity between the sodium ions (Supplementary Fig. 2).

**Fig. 6:**
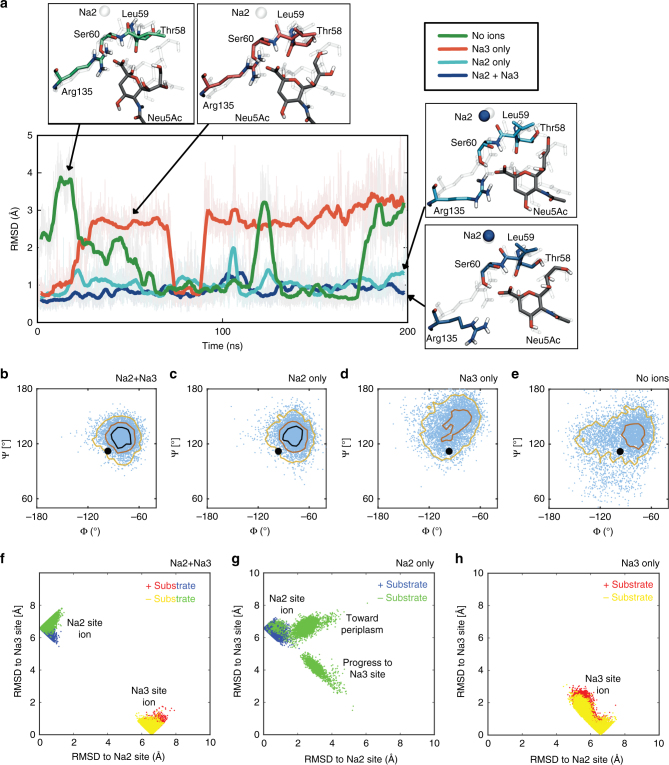
Molecular dynamics simulations of SiaT. **a** Heavy-atom RMSD of the substrate with respect to the X-ray structure for four MD simulations with different combinations of Na^+^ ions in the Na2 and Na3 sites (see legend, upper right). Snapshots compare the instantaneous configuration of Neu5Ac, Arg135, Na2 (when present), and unwound residues in Tm1 (Thr58, Leu59 and Ser60) with the X-ray structure (ghost). Solid lines are smoothed over an 800 ps window, while the original full data set saved every 40 ps is transparent. **b**–**e** Dependence of Leu59 backbone motion on Na^+^ ion occupancy. ϕ and ψ angles for Leu59 from all four simulations with Neu5Ac bound and different combinations of Na^+^ ions in the Na2 and Na3 sites. Both Na2 and Na3 bound (**b**), Na2 bound only (**c**), Na3 bound only (**d**) and no ions bound (**e**). In each panel, the instantaneous angle pairs are plotted every 40 ps over the entire 200 ns simulation (light blue), and the data set is contoured at values of 100 (black), 50 (red) and 10 (yellow). The ϕ and ψ values in the X-ray structure are represented as a black dot. The distributions in panels **d** and **e**, which lack an ion in the Na2 site, are so broad that the black high-density contour does not exist. **f**, **g** Ion stability in the Na2 and Na3 sites. Simultaneous distance of bound Na^+^ ions to the Na2 site and the Na3 site from 200 ns MD simulations in the presence or absence of Neu5Ac with both Na^+^ ions bound (**f**), only the Na2 ion bound (**g**) and only the Na3 ion bound (**h**). In all panels, every point represents a simulation frame saved every 40 ps, the Na2 ion position is blue (with substrate) or green (without substrate), and the Na3 ion position is red (with Neu5Ac) or yellow (without Neu5Ac)

To further explore the influence of Na^+^ binding on the structure of SiaT, we performed eight MD simulations starting from eight permutations of the structure with or without substrate and Na^+^ ions in their identified binding sites (Supplementary Table 2). With ions in both Na sites, Neu5Ac is stably bound within the binding pocket over the 200 ns timescale maintaining its hydrogen bonds with the unwound section of Tm1 (Fig. 6a). Specifically, the carbonyl oxygen of Leu59 directly interacts with the Na2 Na^+^, which stabilises the neighbouring Ser60 so that it can maintain a bidentate interaction with the carboxyl oxygen atoms of Neu5Ac, as observed in the structure (Supplementary Movie 2). Additionally, the other neighbouring Thr58 residue is able to maintain a backbone hydrogen bond to the hydroxyl group at the C2 position of Neu5Ac. In contrast, removing Na^+^ from the Na2 site makes Neu5Ac unstable with the root mean squared deviation increasing to 3–4 Å (Fig. 6a). The dihedral angles that Leu59 adopts are much greater (Fig. 6b–e), indicating that the Na2 site influences the protein flexibility in the vicinity of the substrate binding site.

For Na2-only simulations, the ion is stable in the site in the presence of substrate, but becomes more mobile in its absence (Fig. 6f–h). In fact, the Na2 ion transitions towards the Na3 site coming within 2 Å of the deeper site, while concomitantly moving 5–6 Å away from Na2. Ion distributions in the Na3-only simulations are independent of substrate occupancy and show moderate localisation to the Na3-site identified in the structure, with a slight tendency to move toward the Na2-site. We do not observe bulk Na^+^ enter the empty Na2/Na3 sites, nor do we observe water permeate the transporter, unlike simulations of the inward-facing structure of vSGLT^39^.

### Alternating access

**Fig. 7:**
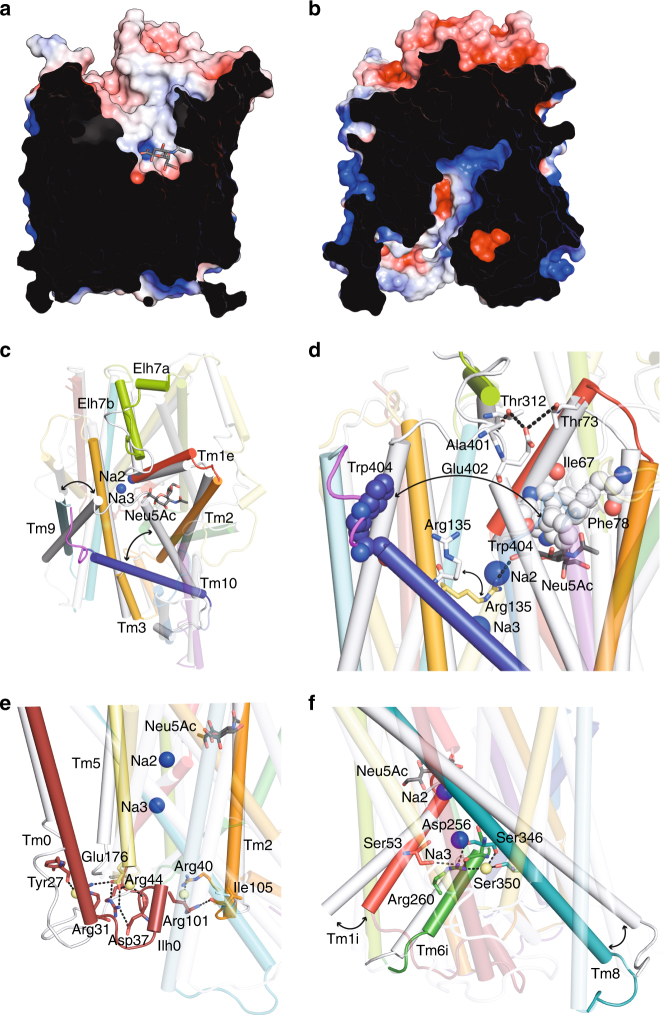
The outward open structure and an inward open model of SiaT. **a**, **b** Surface representation of the outward open structure (**a**) and the inward open model (**b**). Neu5Ac is shown in grey sticks coloured by atom type. Positive potential is shown in blue and negative potential in red. **c**, **d** Predicted movement of transmembrane helices between the outward open structure (coloured) and the inward open model (white) at the periplasmic side. Transmembrane helices implicated in the conformational change between states are labelled. Neu5Ac is shown in grey sticks coloured by atom type and the Na^+^ ions are depicted as blue spheres. Hydrophobic gate residues are depicted as spheres coloured by atom type (**d**). **e** Movement of transmembrane helices between the outward open structure (coloured) and the inward open model (white) at the cytoplasmic side. The cap helix (Ilh0), transmembrane helices and amino acids implicated in opening and stabilising the inner gate are highlighted. **f** Transmembrane helices and amino-acid interactions that stabilise the opening of the gate at the cytoplasmic side are highlighted

To explore the transport mechanism, we constructed an inward-facing model of SiaT based on vSGLT^16^ and created morphs between both states (Fig. 7, Supplementary Movies 3 and 4). Starting from the outward-facing state, the outer gate closes over the binding pocket through a large ~17 Å movement of the N-terminus of Tm10 towards Tm1e and Tm2, which concomitantly moves Tm9 ~10 Å towards Tm3. Additionally, the extracellular loop helices (Elh7a and Elh7b) collapse into the extracellular vestibule and form contacts with the central portion of Tm1e (Fig. 7c, d), as observed in LeuT and Mhp1^40,41^. The outer gate is stabilised in the closed position by hydrogen bonds between Ala401 and Glu402 in the Tm9-Tm10 loop and Thr312 (Elh7) and Thr73 (Tm1e), respectively. Upon closure, an outer gate comprised of hydrophobic residues is created above the substrate-binding site composed of Trp404 (Tm9-Tm10 loop), Ile67 (Tm1) and Phe78 (Tm2) (Fig. 7d).

Substantial movements are also associated with opening the inner gate. The first intracellular loop/helix (Ilh0) between Tm0 and Tm1 is originally in contact with the short intracellular loop between Tm4i and Tm5 occluding the substrate from the cytoplasm (Fig. 7e). The contact is stabilised by salt bridges between the two conserved Arg31 and Arg44 (Ilh0) with Glu176 (Tm4i-Tm5 loop). Ilh0 is further stabilised in this intracellular closed conformation by interactions between Arg40 (Ilh0) and Arg101 and Ile105 (Ilh2a). Interestingly, Arg40 aligns with Arg5 in LeuT, which was previously implicated in intracellular gating^40^. To open the inner gate, Ilh0 breaks all of its bonding partners and unravels while moving radially away from the central axis of the transporter. At the same time, the cytoplasmic ends of Tm8 and Tm9 move radially away from the inner pore axis (Supplementary Movies 3 and 4).

Arg260 (Tm6) located just below Na3 also stabilises the inner gate in a closed conformation through interactions with Ser53 (Tm1), Asp256 (Tm6) and Ser346 (Tm8) (Fig. 7f). It is highly conserved among SLC5 members and aligns to Tyr265 in LeuT, which was also implicated in inner gate closure^20^. The Arg260Glu mutant exhibits no uptake (Supplementary Fig. 3), and we speculate that the positive side chain may also block premature Na^+^ exit from the Na3 site prior to adopting the inward-facing state.

We analysed all SSS sequences that contained the primary Na2 site (21,467) to determine the degree of conservation of the Na3 site, allowing for threonine at either Ser345 or Ser346 (Table 2, Supplementary Data 1). Na3 is present in 19.6% (4212) of these sequences including hSGLT1, which transports two Na^+^, but not vSGLT or hSGLT2, which transport only one Na^+^ (Table 2). None of the structures in the closely related neurotransmitter sodium symporter (NSS), betaine/choline/carnitine (BCC) or nucleobase-cation-symport (NCS1) families have the Na3 site—the corresponding residues are all hydrophobic in LeuT^20^, Mhp1^24^, dDAT^42^ and the serotonin transporter (SERT)^43^.

**Table 2:** Sodium to substrate stoichiometry and residues of the sodium Na3 site

Transporter	SiaT	vSGLT^16,25^	hSGLT1^26^	hSGLT2^26^	NIS^28^	EcPutP^27^	BetP^18^	LeuT^20,78^	SERT^43,79^	DAT^21,80^	Mhp1^24^
Family	SSS	SSS	SSS	SSS	SSS	SSS	BCC	NSS	NSS	NSS	NCS1
Pdbid^a^	5NV9	3DH4	ND	ND	ND	ND	4LLH	4MM4	5I6X	4M48	2JLN
Side chain	**D182** ^**b**^	**D189**	**D204**	**D201**	**D191**	**D187**	S306	A195	V281	V265	N168
Main chain	**S342**	**S364**	**S392**	**S392**	**S353**	**S340**	**T467**	**T354**	D437	D420	S312
Side chain	**S345**	A367	**T395**	A395	**S356**	**S343**	D470	I357	F440	F423	P315
Side chain	**S346**	**S368**	**S396**	**S396**	**T357**	C344	**S471**	A358	A441	G424	A316
Side chain	**T57** ^**c**^	A63	**S77**	**S74**	**S66**	**S54**	A148	V23	V97	V45	M39
Stoichiometry	2:1	1:1	2:1	1:1	2:1	1:1	2:1^d^	2:1^d^	1:1^e^	2:1^d^	1:1

*SSS* solute-sodium symporter family, *BCC* betaine/choline/carnitine family, *NSS* neurotransmitter sodium symporter family, *NCS1* nucleobase-cation-symport family

^a^DOI for pdb codes: pdbid: 3DH4, 4LLH, 4MM4, 5I6X, 4M48, 2JLN

^b^Residues that are conserved to SiaT are highlighted in bold

^c^Second coordination shell

^d^2 Na^+^ to 1 substrate, but no Na3 site present

^e^1 Na^+^, 1 Cl^−^ and 1 substrate

## Discussion

Our results suggest that this subgroup of the SSS family utilise the binding energy of a second Na^+^ ion to allosterically stabilise the substrate without directly coordinating it as observed for endogenous ligand binding to LeuT^20^ and inhibitor binding to dDAT^42^ and SERT^43^. The simulations indicate that binding a second ion further pre-organises the binding site to increase substrate binding affinity, and it may play an important role in stabilising the outward-facing conformation. Our results inform how secondary active transporters harness additional energy from ion gradients by changing their stoichiometry, and it might be possible to pharmacologically exploit differences in this mechanism between SSS family members and other transporters with the LeuT fold.

## Methods

### Cloning and mutagenesis

The genes coding for the *Proteus mirabilis* (strain HI4320) sialic acid transporter SiaT (PMI2976) and the *Escherichia coli* sialic acid transporter NanT (P41036) were codon optimised for *E. coli* (GeneArt, ThermoFischer Scientific) (Supplementary Table 3). For crystallisation and functional studies, the gene coding for SiaT was cloned into the pWarf(−) vector^44^ (pSiaT1), which carries a C-terminal human rhinovirus 3C protease (HRV 3C) cleavage site followed by a green fluorescence protein (GFP)-tag and an 8× His-tag. For bacterial growth experiments, the genes coding for SiaT and NanT were cloned into the low-copy vector pJ422-01 (pSiaT2 and pNanT1) containing a T5 promoter. Constructs were generated using the In-Fusion HD Cloning Kit (Clontech). Single-point mutations were introduced in pSiaT1 using the QuikChange (II) Site Directed Mutagenesis Kit (Stratagene) (Supplementary Table 4). The identity of each construct was confirmed by DNA sequencing (Eurofins Genomics).

### Bacterial growth experiment

pSiaT2 and pNanT1 were transformed into the *E. coli* JW3193 *∆nanT* strain [NBRP (NIG, Japan):*E. coli*]^45^ and verified by DNA sequencing (Eurofins Genomics). Cells were harvested from starter cultures grown overnight in Luria-Bertani broth supplemented with Zeocin™ (5 µg/mL), washed three times in M9 minimal media and diluted to an OD_600_ of 0.05. Cell culture (10 µL) were added to a Honeycomb Bioscreen plate (100 wells) containing M9 media (360 µL) supplemented with Zeocin™ (25 µg/mL), isopropyl β-d-1-thiogalactopyranoside (IPTG) (1 mM), thiamin hydrochloride (7 µM) and Neu5Ac (4 mg/mL, 12.9 mM) as the sole carbon source. Growth at 37 °C with shaking at 250 rpm was monitored between 480 and 700 nm using a Bioscreen C automated growth curve analysis system (Oy Growth Curves AB Ltd.) measuring the OD_480-700_ every 20 min. Experiments were carried out in duplicate and with biological triplicates. Growth curves represent the mean of six experiments ± the standard error of the mean (SEM). The *E. coli* BW25113 wild-type strain and JW3193 [NBRP (NIG, Japan):*E. coli*] were used as controls.

### Protein production and purification

The pSiaT1 plasmid was transformed into the *E. coli* Lemo21(DE3) strain (NEB). The strain was grown in Terrific Broth media supplemented with kanamycin (50 µg/mL), chloramphenicol (34 µg/mL), l-rhamnose (250 µM) and induced with IPTG (0.4 mM) at 25 °C overnight with shaking at 200 rpm. Selenomethionine-derivatised (SeMet) protein was produced using PASM-5052 auto-induction media^46^.

Cells were solubilised in phosphate-buffered saline (PBS) supplemented with cOmplete™ EDTA-free protease inhibitor tablets (Roche), lysozyme (0.5 mg/mL), DNaseI (5 µg/mL), MgCl_2_ (2 mM) and disrupted using an EmulsiFlex-C3 (AVESTIN) at 20,000 psi. Cell debris was removed at 24,000×*g*, and the cell membranes were collected with ultracentrifugation at 235,000×*g* for 2 h and stored at −80 °C until further use. Cell membranes were solubilised in 2% (w/v) DDM for 2 h at 4 °C and unsolublised material were removed at 150,000×*g*. The supernatant was subjected to immobilised metal affinity chromatography and loaded onto a 5 mL HisTrap FF column (GE Healthcare) equilibrated with Buffer A (70 mM Tris pH 8.0, 150 mM NaCl, 20 mM imidazole, 6% glycerol, 5 mM β-mercaptoethanol and 0.0174% (w/v) DDM). The protein was purified with an ÄKTA system connected to a JASCO Model FP-2020 Intelligent Fluorescence Detector (excitation: 485 nm and emission 512 nm), washed with Buffer A and collected using a linear gradient up to 75% of Buffer B (Buffer A complemented with 500 mM imidazole) over 30 column volumes. Protein was concentrated and simultaneously exchanged into Buffer C (50 mM Tris pH 8.0, 150 mM NaCl, 5 mM Neu5Ac, 0.0174% (w/v) DDM). The GFP-tag was cleaved with HRV 3C protease in a 1:12.5 mass ratio (enzyme:substrate) at 4 °C for 20 h. Size exclusion chromatography was performed as a final purification step using a HiLoad 16/600 Superdex 200 column in Buffer C. Protein concentration was determined using a ND-1000 spectrophotometer at 280 nm, using the extinction coefficient of 76,445 per M per cm and a molecular weight of 55.1 kDa.

For purification of the SeMet protein, a reverse immobilised metal affinity step was added following the HRV 3C protease cleavage. The sample was passed through a 5 mL His-TRAP FF column equilibrated with Buffer C.

The SiaT mutants were produced in PASM-5052 auto-induction media and purified in the same way as SeMet protein. For MST, ITC and proteoliposome measurements, the protein samples were produced in the same way as mutants and purified without Neu5Ac in Buffer C.

### Crystallisation

Initially, hanging-drop vapour diffusion experiments at 20 °C using a Mosquito nanolitre-dispensing robot were set up using the crystal screens MemGold, MemGold II and MemStart/MemSys. A volume of 0.5 µL protein solution (20 mg/mL) and 0.5 µL reservoir solution were equilibrated over 100 µL of reservoir solution. Crystals of SiaT appeared after 1–2 weeks with reservoir solution composed of 0.1 M sodium citrate pH 5.0, 0.2–0.25 M potassium chloride and 30–40% (w/v) pentaerythritol propoxylate (5/4/PO/OH). SeMet-incorporated crystals were obtained in the same conditions, with 1% OG and 20 mM Neu5Ac added to the reservoir.

### Data collection and structure determination

The native data set was collected at beamline 5.0.2 of The Advanced Light Source (Lawrence Berkeley National Laboratory, Berkeley, CA). The SeMet-SAD data sets were collected at Diamond Light Source at beamline I24. The SeMet-SAD data sets were processed using XDS^47^. SeMet-SAD data sets from 13 different crystals were merged and scaled using BLEND^48^. SeMet sites were identified and refined with the programs SHELX and SHARP^49,50^. The phases were further improved by RESOLVE and an initial model was built using the ARP/wARP web service^51^. The structure from the best-diffracting SeMet-SAD data was determined by PHASER^52^ to 1.95 Å resolution in space group *C*2. The native data set was processed in xia2 through CCP4i and solved to 2.26 Å in space group *P*22_1_2_1_^53,54^. All structures were refined using PHENIX^55^. Data collection and refinement statistics are summarised in Table 1. In X-ray crystallography, it is difficult to differentiate sodium ions from water molecules unless the resolution is under 1.2 Å^56^; however, a distinguishing feature of Na^+^ sites is that they are typically coordinated by 4 to 8 partners at distances less than 2.7 Å^57^. In SiaT, both sites have clear electron density peaks and coordinate five partner atoms at distances ranging from 2.2 to 2.5 Å (Fig. 4a), which is inconsistent with water molecules at these sites.

### Microscale thermophoresis binding assay

Binding assays were carried out on wild type protein using MST performed on a Monolith NT.LabelFree instrument (NanoTemper Technologies). A range of concentrations of Neu5Ac (from 0.3 μM to 10 mM) were incubated with 1 μM of purified SiaT in PBS buffer supplemented with 0.0174% (w/v) DDM for 5 min prior to taking measurements. The samples were loaded into NanoTemper Technologies glass capillaries and MST measurements were carried out using 10% LED power and 40% MST power. The dissociation constants (*K*_d_) were determined using the mass action equation via the NanoTemper Technologies software from duplicate reads of triplicate experiments and reported as ±SEM.

### Isothermal calorimetry

Wild-type protein was concentrated to a final concentration of 77–178 μM using membrane ultrafiltration with a molecular-weight cutoff of 50 kDa. The flow-through was used to dilute a 100 mM stock solution of Neu5Ac to a concentration of 2.5–3.8 mM. A volume of 206 μL of protein was loaded into the sample cell, and 70 μL of Neu5Ac was loaded into the injection syringe. The system was equilibrated to 25 °C with a stirring speed of 750 rpm. Titration curves were initiated by a 1 μL injection followed by 2 μL injections every 180 s. Background corrections were obtained by injection of Neu5Ac into buffer and buffer into protein with the same parameters. The data from triplicate experiments were analysed using ORIGIN 7 with the first injection excluded. The curves were fitted into a single-site binding isotherm. Measurements were made in biological triplicates using a Micro-200 ITC or a PEAQ ITC (MicroCal, Malvern). The *K*_d_ value was reported as ±SEM.

### Reconstitution of SiaT in proteoliposomes

The purified SiaT wild type and mutants were reconstituted by removing the detergent using a batch-wise method^58^. 2.5 μg of protein was mixed with 120 μL 10% C_12_E_8_, 100 μL of 10% egg yolk phospholipids, sonicated to form liposomes. We then added 20 mM of K^+^-gluconate buffered by 20 mM Tris HCl pH 7.0 to create a final volume of 700 μL. The mixture was incubated with 0.5 g Amberlite XAD-4 resin under rotatory stirring (1200 rev/min) at 25 °C for 40 min^59^.

### Transport measurements

After reconstitution, transport experiments were conducted at 25 °C. In brief, 600 μL of proteoliposomes were loaded onto a Sephadex G-75 column (0.7 cm diameter × 15 cm height) pre-equilibrated with 20 mM Tris-HCl pH 7.0, 40 mM sucrose to balance internal osmolarity. To generate a K^+^ diffusion potential, valinomycin (0.75 μg/mg phospholipid) prepared in ethanol was added to the proteoliposomes following Sephadex G-75 column chromatography. As a control, ethanol was added to proteoliposomes, which did not exert any effect on the transport activity. After 10 s of incubation with valinomycin/ethanol, transport was started by adding 50 μM [^3^H]-Neu5Ac to the proteoliposomes in the presence of 10 mM NaCl. The initial rate of transport was measured by stopping the reaction after 10 min, i.e., within the initial linear range of [^3^H]-Neu5Ac uptake into the proteoliposomes. Transport was terminated by removing [^3^H]-Neu5Ac by loading each proteoliposome sample (100 μL) on a Sephadex G-75 column (0.6 cm diameter × 8 cm height). Proteoliposomes were eluted with 1 mL 50 mM NaCl and collected in 4 mL of scintillation mixture, vortexed and counted. Uptake data were fitted in a first-order rate equation for time course plots. Radioactivity uptake in controls performed with liposomes (without incorporated protein) were negligible with respect to transport data. Non-linear fitting analysis was performed by Grafit software (version 5.0.13). To measure the specific activity of SiaT and mutants, the amount of protein was estimated as described in the above sub-section (Protein production and purification). All measurements are presented as means ± SD from five independent experiments.

### Ultracentrifugation of proteoliposomes

To verify proper incorporation of the wild type and mutant SiaT variants into proteoliposomes, reconstitution mixtures were passed through Sephadex G-75 column and 600 µL were ultracentrifuged (110,000×*g*, 1 h, 4 °C). Pellets were solubilised with 3% SDS and subjected to 12% SDS-PAGE and silver stained for detection.

### Molecular dynamics simulations

Eight systems (S1-S8) were simulated starting with different ion and substrate bound conformations of the transporter (Supplementary Table 2). Initially, the protein was oriented in the membrane using the online server Orientation of Proteins in Membranes (OPM)^60^. Titratable states were addressed with PROPKA calculation in the membrane framework with APBSmem, v2.0.2^61^. Next, the transporter was inserted in each of the 8 states described in Supplementary Table 2 into a 1-palmitolyl-2-oleoyl-sn-glycero-3-phosphatidylethanolamine (POPE) membrane using the CHARMM-GUI Membrane Builder^62^. Each system was then solvated in a rectangular box (90 × 90 × 106 Å^3^) containing 150 mM Na^+^ and Cl^−^ resulting in final system sizes of ~86,000 atoms. All system files were then converted from CHARMM to AMBER format with in house scripts. Simulations were carried out using the ff14SB AMBER parameter set for the protein^63^, GLYCAM06 for the Neu5Ac^64^, the Joung-Chetham parameters for the monovalent ions^65^ and LIPID14 for the lipids^66^. The TIP3P model was used to simulate the water^67^. All systems were then minimised with NAMD version 2.10^68^, using conjugate gradient for 10,000 steps. Following minimisation, the systems were gradually heated from 10 to 310 K at a rate of 20 K/15 ps using temperature reassignment. During the heating phase, the dynamics were carried out in the constant volume/temperature (NVT) ensemble, using a 1 fs integration interval and 50 kcal/mol/Å^2^ harmonic restraints on Na^+^ in the Na2 and Na3 sites, the heavy atoms of the Neu5Ac, all protein heavy atoms, and two bound Cl^−^ ions resolved in the structure. The lipid head groups and water oxygen atoms were harmonically restrained with 20 and 2 kcal/mol/Å^2^ force constants, respectively. After reaching 310 K, the force constraints on the water were decreased by half followed by a 25 ps NVT simulation. Next, we switched to the NPT ensemble using the Langevin piston barostat with a 200 fs piston period and 100 fs piston decay constant to maintain the pressure at 1 bar. Temperature was maintained at 310 K using Langevin dynamics with a 0.5/ps damping coefficient. For the next 610 ps, the restraints were reduced to 10 kcal/mol/Å^2^ for the heavy atoms of the protein backbone, ring atoms of the sialic acid, and bound Na^+^ and Cl^−^ atoms; 5 kcal/mol/Å^2^ for the side chain heavy atoms, the terminal substrate heavy atoms, and the lipid head group atoms; and the water restraints were reduced to 0.5 kcal/mol/Å^2^. During the subsequent 610 ps, the force restraints on the protein, substrate, ions, and lipids were decreased by half; the waters were released; and the integration time step was increased to 2 fs. All remaining restraints were then gently reduced over the next 1.8 ns followed by 5 ns equilibration without restraints. Finally, each system was simulated for 200 ns. Hydrogen bond lengths were restrained with the SHAKE algorithm^69^. Each system was neutralised during setup, and the particle mesh Ewald summation method was used to calculate long range electrostatics with the default cubic order interpolation order. All short range interactions were switched to zero at 10 Å.

### Homology modelling of SiaT in inward-open conformation

The inward-facing conformation of SiaT was modelled on the inward-facing structure of the SSS Na^+^/galactose cotransporter from *Vibrio parahaemolyticus* (vSGLT). The two transporters share ~24% of sequence identity and ~46% of sequence similarity. Initially, an alignment between the two proteins was carried out using a global sequence alignment with *EMBOSS stretcher*^70^ followed by a second, independent structural alignment with *MatchMaker*^71^ performed within Chimera (ver. 1.10.1)^72^. Since the inward and outward-facing states adopt distinct configurations, the structural alignment produced suspect results in certain areas specifically around the Tm9–10 region, which undergoes large conformational rearrangements during gating. Therefore, the consensus alignment from both methods were used followed by a few minor hand adjustments in regions that varied, such as where the structural alignment was problematic. Next, Modeller (ver. 9.15) was used to create inward-facing models of SiaT using chain A of vSGLT (pdbid: 3DH4) as a template structure for the final alignment^73^. One hundred models were generated, and the best Discrete Optimised Protein Energy (DOPE) score along with visual inspection of the loops led to the final model we choose to present in the manuscript^74^.

### Figures and sequence alignments

Figures of protein structures were prepared with PyMOL (PyMOL molecular Graphics System; Schrödinger LLC) and Ligplot^+^^75^. All figures were made using the coordinates from the seleno-derived SiaT. Secondary structure was assigned using the program DSSP^76^. Multiple protein sequence alignment was performed between SiaT and additional SiaT sialic acid transporters from *Morganella morganii* (WP_004237805.1), *Salmonella enterica* (KYN56341.1), *Vibrio fischeri* (AAW85163.1), *Plesiomonas shigelloides* (WP_010863240.1), *Photobacterium profundum*(WP_011218958.1), *Staphylococcus aureus* (WP_000665723.1), *Clostridium perfringens* (WP_003457485.1), *Clostridium difficile* (WP_021423455.1) and *Streptococcus pneumoniae* (WP_061771177.1). This alignment was generated using ClustalW, and ESPript 3 with manual editing. To compare conservation of the Na2 and Na3 site, vSGLT (pdbid: 3DH4) and Mhp1 (pdbid: 2JLN) were superposed onto SiaT in PyMOL and then hSGLT1 and hSGLT2, NIS, EcPutP were aligned based on sequence homology using ClustalW, LeuT (pdbid: 4MM4), dDAT (pdbid: 4M48), SERT (pdbid: 5I6X) and BetP (pdbid: 4LLH) were structurally aligned to look at the Na2 conservation. The Na3 site was derived by proximity to the Na2 site.

### Evolutionary conservation of the Na^+^-binding sites

Evolutionary conservation of the Na2 and Na3 sites throughout the SSS family was assessed by downloading all sequences (39,612) in UniProt matching to the HMM profile of the Pfam family PF00474 (SSF) on 2 May 2017. The C2LEL6.1 protein sequence was used as reference to construct a HMM profile using hmmbuild, part of the HMMER3 package version 3.1b^77^ and the sequences representing the Pfam PF00474 family were aligned to this HMM profile using hmmalign. A custom alignment tool (COAT; available from http://microbiology.se/software/coat/) was used to cluster the aligned sequences based on the residues present in the positions of the Na2 site (342, 343) and Na3 site (57, 182, 345, 346) of the SiaT protein sequence. Based on these clusters, the frequencies of conserved residues were established. Out of 39,612 sequences, 21,467 (54.2%) had a conserved Na2 site, of which 4212 (10.6%) also had a conserved Na3 site (Supplementary Data 1). In addition, 45 sequences apparently had a conserved Na3 site, but curiously lacked the Na2 site.

### Data availability

Coordinates and structure factor files have been deposited to the Protein Data Bank (PDB) under the accession numbers 5NV9 (SeMet) and 5NVA (native). Other data are available from the corresponding authors upon reasonable request.

## Electronic supplementary material

Supplementary Information

Description of Additional Supplementary Info

Supplementary Movie 1

Supplementary Movie 2

Supplementary Movie 3

Supplementary Movie 4

Supplementary Dataset 1
