# Supplementary material for: Substrate-bound outward-open structure of a Na+-coupled sialic acid symporter reveals a new Na+ site
Source: Nat Commun. 2018 May 1;9:1753. doi: 10.1038/s41467-018-04045-7 (PMC5931594; doi:10.1038/s41467-018-04045-7)
Supplement: Supplementary file 1 — Supplementary Information [file 41467_2018_4045_MOESM1_ESM.pdf]

Substrate-bound outward-open structure of a Na^+-coupled sialic acid symporter reveals a new Na^+ site

Wahlgren et al.

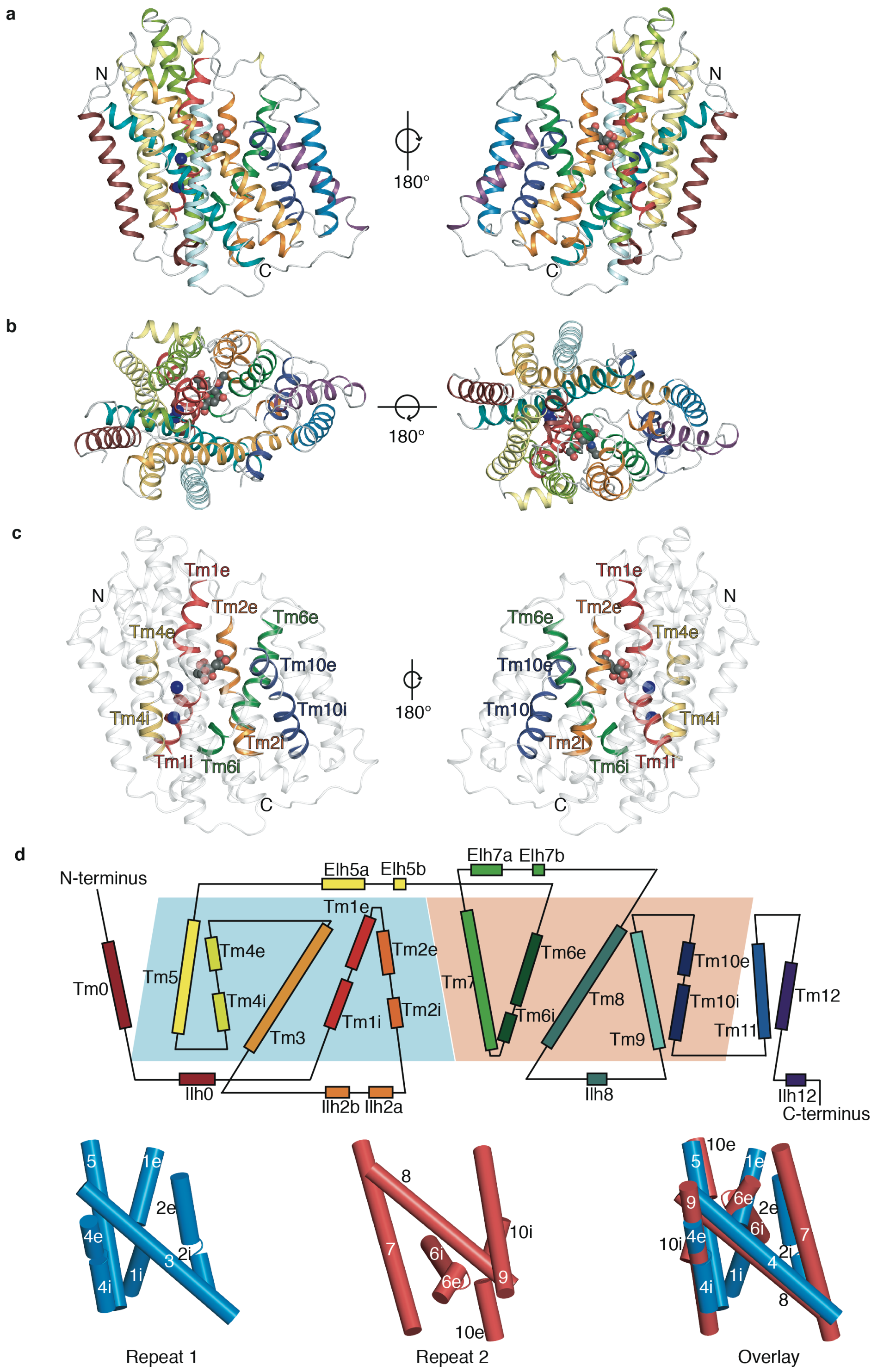

Supplementary Fig. 1 The crystal structure of SiaT. **a** Side-view of SiaT in the membrane plane, with Neu5Ac (grey spheres, coloured by atom type) and Na^+ ions (blue spheres) bound. **b** Top-view (left) of SiaT viewed from the periplasmic side of the membrane bilayer and bottom-view (right) of SiaT viewed from the cytoplasmic side of the membrane bilayer. **c** Unwound transmembrane helices depicted in colour, while the remaining helices are coloured in white. **d** The topology of SiaT represents an inverted repeat made up of five

transmembrane helices in each. The blue and red trapeziums represent the inverted topology of Tm1 to Tm5 and Tm6 to Tm10, respectively. The two inverted repeats are related by an apparent two-fold symmetry around an axis through the centre of the membrane plane. A superposition of SiaT Tm1 to Tm5 (blue) with Tm6 to Tm10 (red) yields an RMSD of 6.6 Å for 75 Cα atoms. Intracellular and extracellular loops are not shown.

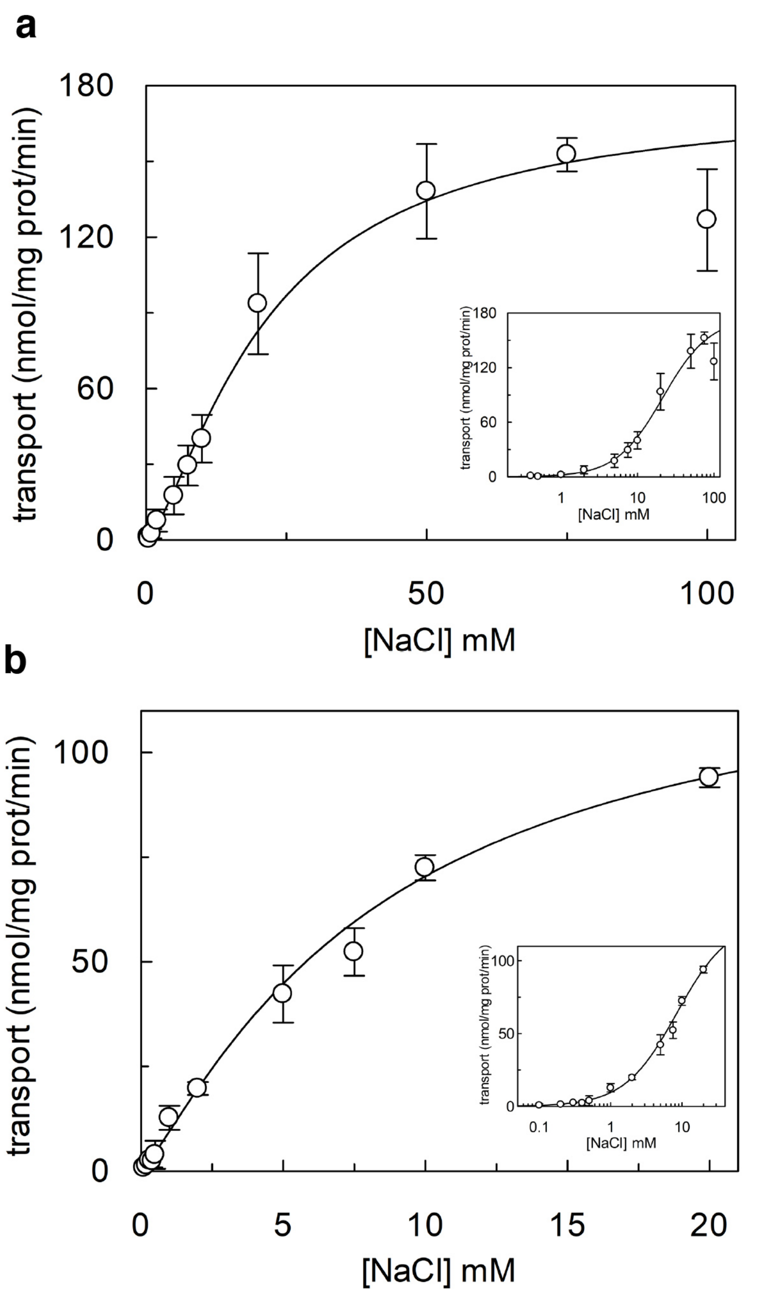

Supplementary Fig. 2 Determination of the SiaT Na^+ Hill coefficients for Ser345Ala (**a**) and Ser346Ala (**b**) SiaT. Data were plotted using the Hill equation. The insets show the low concentration data points. Data are presented as means ± SD from five independent experiments.

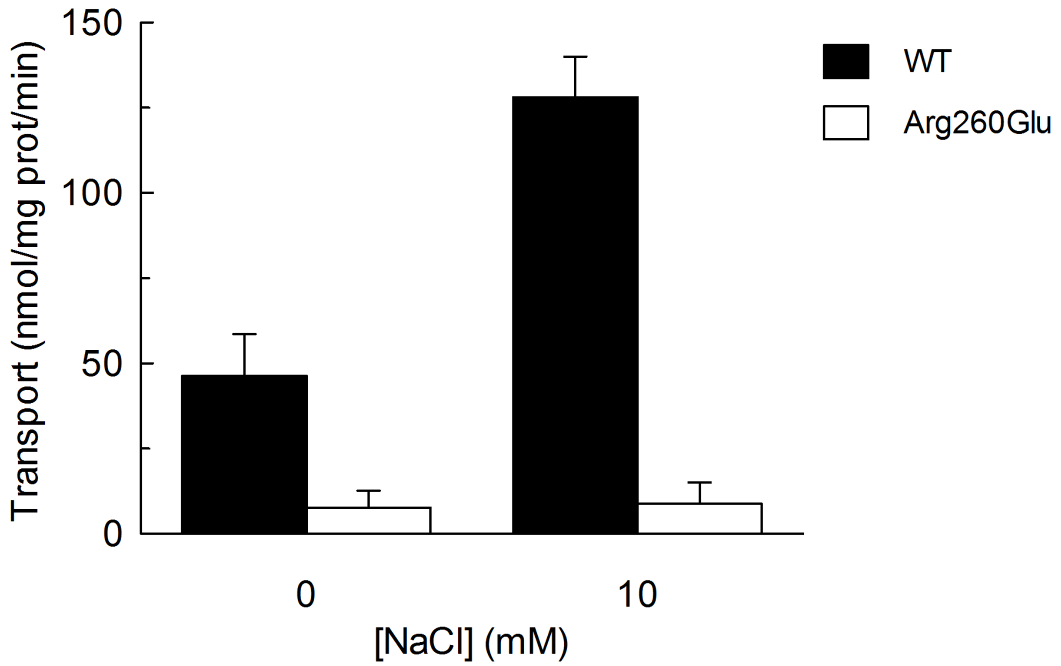

Supplementary Fig. 3 The Neu5Ac transport by SiaT and mutant Arg260Glu. The transport of [^{3}H]-Neu5Ac in the presence of 10 mM NaCl was measured in proteoliposomes reconstituted with wild type SiaT (wildtype) and the mutant Arg260Glu with an imposed K^+ diffusion membrane potential. All proteoliposome measurements are presented as means ± SD from five independent experiments.

Supplementary Table 1 Predicted and characterised sialic acid transporters of the SSS-type in pathogenic bacteria

Genbank Accession number	Organism	E-value	Identities/ Positives (%)	Infection
WP_036933635.1	*Proteus vulgaris*	0	96/98	Nosocomial infections
WP_064718270.1	*Proteus hauseri*	0	95/97	Nosocomial infections
EEG86339.1	*Proteus penneri ATCC 35198*	0	95/98	Nosocomial infections
WP_004237805.1	*Morganella morganii*	0	92/96	Nosocomial infections
KYN56341.1	*Salmonella enterica subsp. enterica serovar* Typhimurium[a,10]	0	83/92	Typhoid fever
WP_000628084.1	*Salmonella enterica*	0	83/92	Salmonellosis
WP_010863240.1	*Plesiomonas shigelloides*	0	71/85	Gastroenteritis
WP_000665723.1	*Staphylococcus aureus*	4E-139	46/67	Staph infections
WP_021423455.1	*Clostridioides difficile*	6E-91	34/57	Nosocomial infections
WP_061771177.1	*Streptococcus pneumoniae*	2E-84	34/57	Pneumococcal infections

[a]Characterised SiaT transporter

Supplementary Table 2 Ion and substrate occupancies for eight distinct simulated systems

Binding Site	S1	S2	S3	S4	S5	S6	S7	S8
**Na2**	√[a]	√			√	√		
**Na3**	√		√		√		√	
**Neu5Ac**	√	√	√	√				

[a]Check marks indicate that Na^+ or Neu5Ac is present in the structure at the start of the simulation.

Supplementary Table 3 Codon optimized genes

	Sequence 5' - 3'
SiaT (PMI2976)	ATGCAACTGCATGATTTTGGCTTTATTAACTATGCCGTGCTGTTTGGTTATCTGGCAGCAATGCTGCTGGTTGGTGTGTA<br>TTTTAGCAAACGTCAGAAAACCGCAGATGATTATTTTCGTGGTGGTGGTCGTGTTCCGGGTTGGGCAGCCGGTGTTAGCG<br>TTTTTGCAACCACCCTGAGCAGCATTACCTTTATGAGCATTCCGGCAAAAGCATATACCAGCGATTGGACCTTTATTATC<br>GGTCAGTATCTGGCCATTGCAATTCTGCCGCTGGTGTTTTATTTCTATATTCCGTTTTTTCGCAAACTGAAAATCACCAG<br>CGCATATGAATATCTGGAAGCCCGTTTTGATGTTCGTAGCCGTCTGTTTGCAAGCCTGAGCTTTATGCTGTTTCATATTG<br>GTCGTGTGGCCATTATTACCTATCTGACCGTTCTGGCACTGCGTCCGTTTATGGGTATTGATCCGGTTGTTCTGATTGTG<br>CTGATTAGCCTGCTGTGTATTATCTATACCTGGATGGGTGGTATTGAAGGTGTTATTTGGACCGATGTTATTCAGGGTCT<br>GCTGCTGAGCGGTGGTGCCGTTCTGATTTTTATCATGATTTGCTTTAAAGTGGACGGTGGCATCAGCGAAATCTTTACCA<br>CCACCGCACAGGCAGACAAATTTTTCCCGACCACCCAGTGGCGTTGGAGCTGGACCGATAGCACCATTCCGGTTCTGATG<br>ATTGGTTTTCTGTTTGCCAATATCCAGCAGTTTACCGCAAGCCAGGATGTTGTTCAGCGTTATATTGTTACCGACAGCAT<br>CAAAGAAACCAAACGTACCCTGATTACCAATGCAAAACTGGTTGCAATTATCCCGATCTTTTTTTCGCCATTGGTAGCG<br>CACTGTTTGTCTATTATCAGCAGAATCCGAGCCTGCTGCCTGCAGGTTTTAATACCGGTGGTATCCTGCCGCTGTTTATT<br>GTGACCGAAATGCCGATTGGTATTGCCGGTCTGATTATTGCAGCAATTTTTGCAGCAGCACAGAGCAGTATTAGCAGCAG<br>TCTGAATAGCATTAGCAGCTGTTTTAACAGCGATATTTACACCCGTCTGAGCAAAAGCAGCCCGAGTCCGGAACAGAAAA<br>TGAAAGTTGCCAAACTGGTTATTATTGTGGCAGGCATTTTTAGCAGCCTGGCAGCCATTTGGCTGGTTCTGAGTGATGAA<br>GCAGAAATTTGGGATGCATTTAATAGCCTGATTGGTCTGATGGGAGGTCCGATGACCGGTCTGTTTATGCTGGGCATTTT<br>TGTTAAACGTGCAAATGCAGGTAGCGCAGTTGTTGGTATTATTGTTAGCATTATTGCGGTTCTGGCAGCGCGTTATGGTA<br>GCGATCTGAATTTTTTCTTTTATGGCGTGATTGGTAGCATGAGCGTTGTTATTGCAGGCACCATTACCGCACCGCTGTTT<br>GCACCAGCGAAACAGCTGAGCCTGGATGATAGCGAAACCAGTGAAAAT
NanT (P41036)	ATGAGCACCACCACCCAGAATATTCCGTGGTATCGTCATCTGAATCGTGCACAGTGGCGTGCATTTAGCGCAGCATGGCT<br>GGGTTATCTGCTGGATGGTTTTGATTTTGTTCTGATTGCACTGGTTCTGACCGAAGTTCAGGGTGAATTTGGTCTGACCA<br>CCGTTCAGGCAGCAAGCCTGATTAGTGCAGCATTTATTAGCCGTTGGTTTGGTGGTCTGATGCTGGGTGCAATGGGTGAT<br>CGTTATGGTCGTCGTCTGGCAATGGTTACCAGCATTGTTCTGTTTAGTGCAGGCACCCTGGCATGTGGTTTTGCACCGGG<br>TTATATTACCATGTTTATTGCCCGTCTGGTTATTGGTATGGGTATGGCAGGCGAATATGGTAGCAGCGCAACCTATGTTA<br>TTGAAAGCTGGCCGAAACATCTGCGTAATAAAGCAAGCGGTTTTCTGATTAGCGGTTTTAGCGTTGGTGCAGTTGTTGCA<br>GCACAGGTTTATAGCCTGGTTGTTCCGGTTTGGGGTTGGCGTGCACTGTTTTTTATCGGTATTCTGCCGATTATCTTTGC<br>ACTGTGGCTGCGTAAAAACATTCCGGAAGCAGAAGATTGGAAAGAAAAACATGCAGGTAAAGCACCGGTTCGTACCATGG<br>TTGATATTCTGTATCGTGGTGAACATCGTATTGCCAATATTGTTATGACCCTGGCAGCAGCAACCGCACTGTGGTTCTGT<br>TTTGCAGGTAATCTGCAGAATGCAGCAATTGTTGCCGTTCTGGGTCTGCTGTGTGCAGCAATCTTTATTAGCTTTATGGT<br>TCAGAGCGCAGGTAAACGTTGGCCGACCGGTGTTATGCTGATGGTTGTTGTGCTGTTTGCATTTCTGTATAGTTGGCCGA<br>TTCAGGCACTGCTGCCGACCTATCTGAAAACCGATCTGGCATATAATCCGCATACCGTTGCAAACGTGCTGTTTTTTTCA<br>GGTTTTGGTGCAGCAGTTGGTTGTTGCGTTGGTGGTTTTCTGGGTGATTGGCTGGGCACCCGTAAAGCATATGTTTGTAG<br>CCTGCTGGCAAGCCAGCTGCTGATTATTCCGGTTTTTGCAATTGGTGGTGCAAATGTTTGGGTGCTGGGCCTGCTGCTGT<br>TCTTTCAGCAGATGCTGGGCCAGGGTATTGCAGGTATCCTGCCGAAACTGATTGGTGGTTATTTTGATACCGATCAGCGT<br>GCAGCAGGTCTGGGTTTTACCTATAATGTTGGTGCACTGGGTGGCGCACTGGCACCGATTATTGGTGCCCTGATTGCCCA<br>GCGTCTGGATCTGGGCACCGCACTGGCCAGCCTGAGCTTTAGCCTGACCTTTGTTGTTATTCTGCTGATTGGTCTGGATA<br>TGCCGAGCCGTGTTCAGCGTTGGCTGCGTCCGGAAGCCCTGCGTACCCATGATGCAATTGATGGTAAACCGTTTAGCGGT<br>GCAGTTCCGTTTGGTAGCGCAAAAAATGATCTGGTTAAAACCAAAAGCTGA

Supplementary Table 4 Primer sequences

	Sequence 5' - 3'
pSiaT1_forward	AAGAAGGAGACTCGAGATGCAACTGCATGATTTTGG
pSiaT1_reverse	AGACTTCCAAGGATCCATTTTCACTGGTTTCGCTATC
pSiaT2_forward	TAGGAGGTAAACATAATGCAACTGCATGATTTTGG
pSiaT2_reverse	GGCCTGTACAGAATTTCAGTGGTGGTGGTGGTG
pNanT1_forward	TAGGAGGTAAACATAATGAGTACTACAACCCAGAA
pNanT1_reverse	GGCCTGTACAGAATTTTAACTTTTGGTTTTGACTAAAT
T58A_forward	GGTAATGCTGCTCAGGGCGGTTGCAAAAACGCTAA
T58A_reverse	TTAGCGTTTTTGCAACCGCCCTGAGCAGCATTACC
S60A_forward	CGTTTTTGCAACCACCCTGGCCAGCATTACCTTTATGAGC
S60A_reverse	GCTCATAAAGGTAATGCTGGCCAGGGTGGTTGCAAAAACG
T63A_forward	CAACCACCCTGAGCAGCATTGCCTTTATGAGCATT
T63A_reverse	AATGCTCATAAAGGCAATGCTGCTCAGGGTGGTTG
Q82D_forward	GATTGGACCTTTATTATCGGTGATTATCTGGCCATTGCAATTCTG
Q82D_reverse	CAGAATTGCAATGGCCAGATAATCACCGATAATAAAGGTCCAATC
R135E_forward	CTGAGCTTTATGCTGTTTCATATTGGTGAGGTGGCCATTATTACCTATCTGAC
R135E_reverse	GTCAGATAGGTAATAATGGCCACCTCACCAATATGAAACAGCATAAAGCTCAG
D182A_forward	AGGTGTTATTTGGACCGCTGTTATTCAGGGTCTGC
D182A_reverse	GCAGACCCTGAATAACAGCGGTCCAAATAACACCT
R260E_forward	GCAAGCCAGGATGTTGTTCAGGAGTATATTGTTACCGACAGCATC
R260E_reverse	GATGCTGTCGGTAACAATATACTCCTGAACAACATCCTGGCTTGC
S342A_forward	AATTTTTGCAGCAGCACAGGCCAGTATTAGCAGCAGTCTG
S342A_reverse	CAGACTGCTGCTAATACTGGCCTGTGCTGCTGCAAAAATT
S343A_forward	TTTTGCAGCAGCACAGAGCGCTATTAGCAGCAGTCTGAAT
S343A_reverse	ATTCAGACTGCTGCTAATAGCGCTCTGTGCTGCTGCAAAA
S345A_forward	TGCAGCAGCACAGAGCAGTATTGCCAGCAGTCTGAATAG
S345A_reverse	CTATTCAGACTGCTGGCAATACTGCTCTGTGCTGCTGCA
S346A_forward	AGCACAGAGCAGTATTAGCGCCAGTCTGAATAGCATTAGC
S346A_reverse	GCTAATGCTATTCAGACTGGCGCTAATACTGCTCTGTGCT
