# Supplementary material for: Substrate-bound outward-open structure of a Na+-coupled sialic acid symporter reveals a new Na+ site
Source: Nat Commun. 2018 May 1;9:1753. doi: 10.1038/s41467-018-04045-7 (PMC5931594; doi:10.1038/s41467-018-04045-7)
Supplement: Supplementary file 2 — Description of Additional Supplementary Info [file 41467_2018_4045_MOESM2_ESM.pdf]

Description of Additional Supplementary Files

File Name: Supplementary Movie 1
Description: Overall structure of SiaT. Side-view of SiaT in the membrane plane, with Neu5Ac (grey spheres, coloured by atom type) and two Na+ ions (blue spheres) bound.

File Name: Supplementary Movie 2
Description: Superposition of the Neu5Ac bound simulation with Na+ ions bound in the Na2 and Na3 sites (blue) versus the Neu5Ac bound simulation with no Na+ ions bound (green). The substrate and ions are not shown for clarity. The protein backbones are represented as ribbons, while the residues Thr58, Leu59, Ser60 (unwound segment in middle of movie), and Arg135 (lower middle residue) are represented in sticks with carbon atoms either blue (Na+ ions occupying both the Na2 and Na3 sites) or green (no Na+ ions in the Na2 and Na3 sites). Oxygen atoms are red and hydrogen atoms are white. Leu59 is dynamic in the simulation lacking ions (green), while Arg135 is dynamic in the simulation with both ions bound (blue).

File Name: Supplementary Movie 3
Description: Morph of outward open structure and inward open model viewed from the periplasmic side. Hydrophobic gating residues are shown as spheres and the outer gate residues are shown as sticks. Transmembrane helices implicated in the conformational change are labelled.

File Name: Supplementary Movie 4
Description: Morph of outward open structure and inward open model viewed through the membrane plane. The cap helix (Ilh0) at the cytoplasmic side and transmembrane helices implicated in the conformational change are labelled. Residues involved in interactions that stabilise the cap are shown as sticks.

File Name: Supplementary Data 1
Description: Proteins in UniProt belonging to the SSS family (represented by the SSF Pfam domain) that had a conserved Na2 site and a Na3 site (4,212 in total). The amino acids in the positions corresponding the Na2 (342, 343) and Na3 (57, 182, 345, 346) sites in the P. mirabilis SiaT sequence are given in the table, as well as the region of each protein matching the Pfam SSF domain.
